# The Association between Social Isolation and Physical Activity among Korean Adolescents

**DOI:** 10.3390/children11081003

**Published:** 2024-08-16

**Authors:** Sarang Jang, Na-Young Park

**Affiliations:** 1 Department of Public Health, Sahmyook University, Seoul 01795, Republic of Korea; srjang@syu.ac.kr; 2 Korea Institute for Health and Social Affairs, Sejong 30147, Republic of Korea

**Keywords:** physical activity, social isolation, types of social isolation, adolescent, friendship network

## Abstract

Objectives: This study investigated the impact of structural isolation within peer relationships on physical activity levels among Korean adolescents, exploring how different types of social isolation within classroom networks influence physical activity. Methods: This study utilized cross-sectional survey data. Specifically, using data from the 8th Korean Children and Youth Happiness Index survey (2016), which included 3356 middle and high school students in Korea, the study employed binary logistic regression and social network analysis to assess the relationship between social isolation and physical activity. Based on types of isolation, adolescents were categorized into social avoidant, actively isolated and socially indifferent groups. Results: The analysis demonstrated significant differences in physical activity based on social isolation status (χ^2^ = 13.0, *p* < 0.001) and types of social isolation (χ^2^ = 18.3, *p* < 0.001). Key variables such as gender, household subjective economic status, and self-rated health significantly influenced physical activity. The number of physically active friends had a considerable impact on both non-isolated groups (OR = 1.31, *p* < 0.001) and the actively isolated group (OR = 1.42, *p* < 0.05). The actively isolated group exhibited the highest explanatory power in the logistic regression models (Nagelkerke R² = 0.230). Conclusions: This study has highlighted that not only social isolation but also the type of isolation significantly influences physical activity among adolescents. Understanding these distinctions and tailoring interventions accordingly are crucial for promoting physical activity among socially isolated adolescents.

## 1. Introduction

Considering the significant role of physical activity among adolescents in affecting health outcomes, such as obesity, diabetes, and the prevention of cardiovascular diseases in adulthood [1,2,3,4], there is a need to promote physical activity among this demographic. It not only contributes to physical health but also enhances aspects of mental health, such as self-esteem and body image [5]. It is also associated with long-term health benefits that extend into adulthood [3]. However, according to recent reports by the World Health Organization (WHO)), over 80% of adolescents aged 11 to 17 years do not engage in at least one hour of physical activity daily [6]. This report highlights a gender gap in which girls are generally less active than boys, a discrepancy that has persisted over time [6].

Studies on the impact of peer relationships on physical activity among adolescents demonstrate that peers play a significant role in promoting physical activity [7,8]. Peer influence not only contributes to a positive attitude towards sports but also significantly increases physical activity among adolescents [8]. Recent studies have reported that peer support is more strongly associated with physical activity than parental support [9], suggesting that peers may have a more direct influence on the active behaviors of adolescents.

A substantial body of research has focused on the psychological and emotional aspects of friendships among adolescents, emphasizing support, bonding, and attitudes towards friends [7,8,9]. With growing interest in adolescent friend networks, recent studies have reported on the status within peer relationships and its impact on physical activity. These studies revealed that adolescents tend to engage in physical activities similar to those of their friends, demonstrating strong peer influence within these networks [10,11]. A systematic review examining the influence of adolescent friend networks on health behaviors, including physical activity, highlighted the crucial role that friends play in shaping health behaviors. Notably, adolescents with active friends were more likely to participate in physical activities [12]. Most studies on adolescent behavior with regards to social status have focused on popular friends [13,14,15].

However, studies on physically active behaviors among socially isolated adolescents, who can be considered to be structurally disadvantaged in terms of friendship relationships, are scarce. Social isolation is defined as the physical, social, or psychological separation of an individual or group, characterized by limited social contact and communication [16]. Socially isolated individuals are at higher risk of developing suicidal impulses [17,18] and depression [19,20]. Adolescence is characterized by physical changes and the establishment of new identities and lifestyles. The formation and expansion of various social relationships during this phase act as psychological resources to cope with rapid changes, with peers playing a crucial role as sources of information and influence [21]. Previous studies report that isolation from adolescent friendships can lead to a lack of psychological resources, which not only affects mental health but also increases the likelihood of cardiovascular diseases in adulthood [21].

Social isolation can be multifaceted. It can be classified into different types, such as emotional isolation, social loneliness, and structural isolation. Emotional isolation refers to a lack of close emotional bonds with others, while social loneliness denotes a broader absence of social connections. Structural isolation, the focus of this study, involves the lack of integration into existing social networks and the absence of reciprocal friendships [22]. This form of isolation is particularly concerning as it often results from systemic issues within the social environment, making it harder for isolated individuals to form or maintain friendships.

Studies have shown that adolescents who report feeling lonely and having fewer friends tend to have lower rates of physical activity [23,24,25,26] and are more inclined towards sedentary behaviors requiring minimal movement [27,28]. The rate of practicing of physical activity among socially isolated adolescents varies not only with the physical environment but also with identity, social status, and social relationships [29,30]. Adolescents spend considerable time with their friends at school, and the behaviors of friends or friendship groups significantly impact their physical activity [31]. Various influences, such as peer pressure, shared norms within friendship groups, and friend selection, can affect adolescent health behaviors [30,32]. Therefore, the consideration of friendships is essential for developing interventions for physical activity during adolescence.

Isolated adolescents may have different motivations for health behaviors than those with friends. While some studies suggest that isolated adolescents are less exposed to unhealthy behaviors, such as smoking and alcohol consumption, due to reduced peer access [33], others indicate that isolated adolescents might engage more actively in unhealthy behaviors to alleviate the anxiety and loneliness caused by isolation [34,35]. Existing studies often conflate isolation with the emotion of loneliness [33], strictly apply it to complete isolation [35], or narrowly define it with insufficient consideration of external friendships. Such inconsistent findings regarding isolation and health behaviors may stem from a failure to comprehensively differentiate the dimensions of isolation.

Isolation within adolescent friendship networks constitutes a distinct social status. The degree of connectivity through friend nominations within these networks can assist in classifying multidimensional types of social isolation. The type of isolation influences adolescents’ health behavior motivations and outcomes differently. For instance, complete isolation, in which individuals neither nominate others as friends nor are recognized as friends by their peers, is associated with high levels of anxiety and depression [36]. However, adolescents who do not nominate their peers as friends but are nominated by others, despite spending time in solitude, show no differences in social cognition and competencies compared with their socially active peers [36]. Conversely, those who nominate friends but are not reciprocally nominated experience stress due to peer rejection and are more likely to exhibit unhealthy behaviors to alleviate stress or gain attention [32]. Actively isolated adolescents may be excluded from normative socialization, but they are more likely to participate in physical activities as part of their efforts to assert themselves and form social relationships in an active manner [9]. On the other hand, disinterested or socially avoidant adolescents are more likely to lack social cohesion and experience internalized loneliness, which can result in lower levels of physical activity [9,37]. Considering multidimensional types of isolation assists in clarifying the impact of adolescent social isolation on health behaviors by solidifying the structural status within peer relationships. This understanding assists in classifying high-risk isolation types based on health behaviors, such as physical activity, thereby facilitating the selection of target groups for adolescent health policies and the development of effective strategies.

Adolescence is a developmental stage significantly influenced by peers. To develop effective adolescent health promotion policies, an understanding of health behaviors based on peer relationships is required. Considering the severity of health issues associated with social isolation, priority must be placed on understanding the impact of isolation on health behaviors within peer relationships. However, studies on adolescents who were socially isolated due to relationships rather than physical isolations, such as those caused by the coronavirus disease 2019 pandemic, are scarce and generally limited to specific health behaviors, such as alcohol consumption and smoking [33,35,36,38,39].

Compared to the international community, the decrease in the rates of physical activity and the increase in sedentary behaviors are severe among Korean adolescents. According to a recent WHO report [6], over 90% of Korean adolescents engage in less than one hour of physical activity per day, with females showing the lowest rates globally. To effectively address this issue, efforts must be made at the individual and institutional levels to formulate strategies that consider interpersonal differences. For instance, it is necessary to identify motivations for health behaviors in isolated adolescents who are at high risk and intervene proactively. The objectives of this study were twofold: first, to investigate the effects of structural isolation on physical activity within peer relationships, and second, to analyze the impacts of various types of isolation on physical activity within classroom friend networks.

## 2. Materials and Methods

### 2.1. Participants and Procedures

This study used data from the 8th Korean Children and Youth Happiness Index survey conducted in 2016. This dataset, collected annually since 2009, is part of a national survey aimed at comparing indices of happiness among Organization for Economic Co-operation and Development (OECD) countries. The methodology employed was that of a cross-sectional study. The 2016 survey data included peer nomination information within classroom settings, allowing the analysis of social networks and physical activity among adolescents.

The dataset included a sample of 7908 students from elementary, middle, and high schools across South Korea, excluding Jeju Island. Participants were selected through proportional probability sampling to ensure that they accurately represented the national adolescent population. The sampling process included stratification by region and school type to enhance representativeness. The survey was conducted in a paper-based format during school hours and administered by trained survey administrators, thereby ensuring consistency and reliability in the responses gathered.

The analysis in this study focused on 3356 middle and high school students who responded to questions regarding their physical activity. Physical activity levels were assessed using a validated questionnaire that asked about the frequency and duration of various physical activities over the past week. Peer nominations were collected by asking students to list their friends within the classroom, providing data on social network structures.

### 2.2. Measures

This study aimed to investigate the impact of social isolation on physical activity in adolescents. Specifically, it analyzed how physical activity differed between socially isolated and non-isolated adolescents in their classroom peer networks. Using nationally representative data from Korean adolescents, a cross-sectional analysis was conducted to examine the influence of loneliness and social isolation on physical activity.

Social network analysis was used to assess classroom peer relationships, deriving the degree centrality to classify types of isolation. Subsequently, a binary logistic regression analysis was performed to compare the factors influencing physical activity according to the refined isolation type. This study is expected to contribute to a better understanding of how social isolation affects the physical activity levels of adolescents and to inform the development of effective intervention strategies.

#### 2.2.1. Dependent Variable

The dependent variable, physical activity, was categorized based on whether adolescents engaged in at least one hour of vigorous physical activity for fewer than three days or three or more days in the previous week. According to the physical activity guidelines of the U.S. Centers for Disease Control and Prevention, it is recommended that children and adolescents participate in at least 60 min of moderate to vigorous physical activity daily for a minimum of three days per week [40]. Considering this guideline, in the present study, respondents who answered that they had engaged in physically vigorous activity for more than one hour on three or more days in the past week were considered to have participated in physical activity.

#### 2.2.2. Social Isolation and Type of Social Isolation

**Figure 1:**
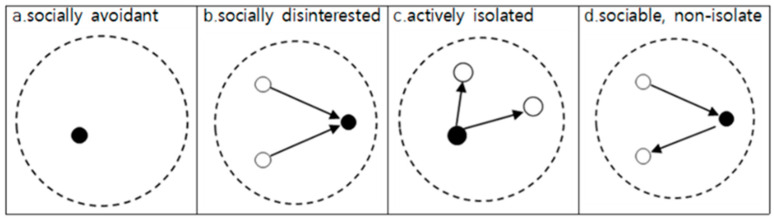
Types of social isolation. Note: Black Circle represents the survey respondent themselves (ego), who interacts with their surroundings (alters) through their social connections in the network analysis; White Circle represents an alter located within the social network of the respondent, ego, considered socially significant by the ego; Arrow represents the relationship between the respondent ego and another actor (friend) whom the ego has nominated or who has nominated the ego, with the direction of the arrow indicating that the actor has been nominated as a friend.

Isolation was classified into isolation status and isolation type. In this study, participants were asked to list up to seven of their closest friends by name or student number, and we reconstructed the classroom friend network data using only those who nominated classmates. Isolation status was determined by the number of close friends in the class; having no close friends was considered isolated, whereas having one or more close friends was considered not isolated. Additionally, multidimensional isolation types were categorized based on a previous study [38] using a combination of in-degree and out-degree nominations within classroom peer relationships (Figure 1).

The first type was socially avoidant isolation (Figure 1a), in which the respondent neither nominated classmates as friends nor was nominated by any classmates (in-degree = 0, out-degree = 0). The second type is socially disinterested isolation (Figure 1b), in which the respondent is nominated by classmates but does not nominate anyone as a friend (in-degree > 0, out-degree = 0). The third type was actively isolated (Figure 1c), where the respondent nominated classmates as friends but was not nominated by anyone (in-degree = 0, out-degree > 0). Finally, sociable (non-isolated) friendships (Figure 1d), a variable contrasting with isolation, involved reciprocal nominations between the respondent and classmates (in-degree > 0, out-degree > 0).

#### 2.2.3. Independent Variables

Gender, grade level, age, subjective economic status, subjective health status, loneliness, and the number of friends engaged in physical activity were included in the analysis model. Gender was categorized as boy or girl, and grade level was classified as middle school or high school. Age was considered a continuous variable. Subjective economic status was measured using the question, “How do you perceive your household’s economic condition?” Responses were given on a 6-point scale (upper upper, upper lower, middle upper, middle lower, lower upper, or lower lower), with higher values indicating a more vulnerable economic status.

Subjective health status was assessed using “I think I am healthy” on a 5-point scale, categorizing “strongly agree” and “agree” as “above average”, while “neutral”, “disagree”, and “strongly disagree” were categorized as “below average”. Loneliness was measured using the question, “I feel extremely lonely without any particular reason,” with responses on a 5-point scale. Those who answered “very true” or “true” were classified as “high loneliness”, while those who answered “not true at all”, “not true”, or “neutral” were classified as “low loneliness.”

The number of friends engaged in physical activity was defined as the number of classmates nominated as friends who reported engaging in at least one hour of physical activity for three days or more per week.

#### 2.2.4. Statistical Analysis

Data analysis was performed using IBM SPSS Statistics 26 software, employing descriptive statistics, chi-square tests, and binary logistic regression analysis. Additionally, to identify multidimensional types of isolation, NetMiner 4 was used to analyze the degree of connections within classroom friendships, classify isolation types, and determine the number of friends engaged in physical activity.

Initially, a cross-sectional analysis was performed to examine physical activity practice, according to isolation status and types of isolation. Furthermore, a social network analysis was performed to investigate the factors influencing physical activity practice by multidimensional isolation types. Using NetMiner 4, the degree of connection between respondents and their classmates were extracted from the classroom friendship network data to classify isolation types. Subsequently, binary logistic regression analysis was performed to assess physical activity practice according to the refined isolation types. The model included variables such as gender, grade level, age, subjective economic status, and subjective health status. The criterion for statistical significance was set at *p* < 0.05.

## 3. Results

### 3.1. General Characteristics of the Participants

This study analyzed data from a nationwide survey targeting South Korean adolescents. A total of 3356 middle and high school students responded, with 47.7% male students (*n* = 1600) and 52.3% female students (*n* = 1756), as shown in Table 1. The ages of the participants were relatively uniformly distributed, ranging from 13 to 18 years. The largest age groups were 15 years (19.5%, *n* = 655) and 16 years (19.8%, *n* = 666), whereas the smallest was 14 years (14.1%, *n* = 472). The participants were almost evenly divided into middle school (49.2%, *n* = 1652) and high school (50.8%, *n* = 1704). In terms of self-rated health, 24.3% (*n* = 817) rated their health as below average (≤3), and 75.6% (*n* = 2536 as above average (≥4). Household subjective economic status was scored from 1 to 6, with lower scores indicating greater wealth, and the mean score was 3.3 (standard deviation [SD] = 0.9). The prevalence of loneliness was 15.7% (*n* = 526), with the majority of the participants (84.2%, *n* = 2826) not feeling lonely. The mean number of physically active friends was 0.50 (SD = 1.0). In terms of social isolation subtypes, 50.5% (*n* = 1695) were categorized as sociable non-isolates, 20.9% (*n* = 701) were socially avoidant, 13.0% (*n* = 435) were socially disinterested, and 15.6% (*n* = 525) were actively isolated. Regarding physical activity, 51.7% (*n* = 1734) engaged in physical activity fewer than three times per week, while 47.0% (*n* = 1577) engaged in physical activity three or more times per week.

### 3.2. Comparison of Physical Activity Based on Social Isolation Status

The association between social isolation status and the frequency of engaging in physical activity for at least one hour, three or more times per week, among Korean adolescents was determined (Table 2). Forty-five participants with missing values for the physical activity-related item were excluded from the analysis; thus, 3311 participants were included in the analysis. Physical activity was categorized into two groups: those engaging in physical activity fewer than three times per week and those engaging in physical activity three or more times per week. Among all participants, 52.4% (*n* = 1734) engaged in physical activity fewer than three times per week, while 47.6% (*n* = 1577) engaged in physical activity three or more times per week. Based on social isolation status, 1678 adolescents were identified as sociable non-isolates and 1633 as isolated. Among the sociable non-isolates, 49.3% (*n* = 827) were less active and 50.7% (*n* = 851) were more active. Conversely, among the isolated adolescents, 55.5% (*n* = 907) were less active and 44.5% (*n* = 726) were more active.

A chi-square test revealed a significant association between social isolation and physical activity levels, with a χ² value of 13.0 (*p* < 0.001), suggesting that isolated adolescents are likely to engage in physical activity less frequently than their non-isolated counterparts.

### 3.3. Comparison of Physical Activity by Types of Social Isolation

Socially isolated adolescents were categorized according to the type of social isolation, and their physical activity levels were compared based on whether they engaged in physical activity for more than one hour at least three times per week (Table 3). The types of social isolation included socially avoidant, socially disinterested, and actively isolated which were compared to a group of individuals who were not socially isolated from the total respondents. Physical activity fewer than three times per week was observed in 52.4% (*n* = 1734) of all adolescents and 49.3% (*n* = 827) of non-isolated adolescents. All three isolation groups had greater than 50% who engaged in physical activity fewer than three times per week: socially avoidant, 58.7% (*n* = 402); socially indifferent, 54.8% (*n* = 235); and actively isolated, 52.0% (*n* = 270). Conversely, physical activity more than three times per week was observed in 50.7% (*n* = 851) of the non-isolated adolescents, whereas it was less 50% in all three isolated groups: socially avoidant, 41.3% (*n* = 283); socially indifferent, 45.2% (*n*=194); and actively isolated, 48.0% (*n* = 249).

A chi-squared test showed a significant association between types of social isolation and levels of physical activity, with a χ² value of 18.3 (*p* < 0.001), suggesting notable variations in patterns of physical activity among the various subtypes of social isolation.

### 3.4. Comparing the Determinants of Physical Activity between Socially Isolated and Non-Isolated Korean Adolescents

The factors influencing physical activity, defined as engaging in physical activity for at least one hour per day, three or more times per week, among those who were socially isolated and those who were not were compared (Table 4). Of the 3311 adolescents, 1678 were not isolated (sociable non-isolates) and 1633 were isolated. The analysis found that girls were less likely to engage in physical activity compared to boys across both groups. The odds ratio (OR) for girls was 0.42 in the entire sample, 0.46 in the non-isolated group, and 0.38 in the isolated group (all *p* < 0.001) indicating a significant gender disparity in physical activity levels. High school students were less likely to engage in physical activity compared to middle school students, particularly in the isolated group (OR = 0.42; 95% confidence interval [CI]: 0.28–0.63; *p* < 0.001), while the difference was not significant in the non-isolated group. Age showed a negative correlation with physical activity in the non-isolated group (OR = 0.78; 95% CI: 0.68–0.89; *p* < 0.001), indicating that older adolescents in this group were less active. However, this trend was not observed in the isolated group. Adolescents who rated their health as above average were significantly more likely to be physically active across all groups (total: OR = 1.69; non-isolated: OR = 1.70; isolated: OR = 1.67; all *p* < 0.001). A lower score indicating greater wealth was associated with increased physical activity only in the isolated group (OR = 0.87; 95% CI: 0.78–0.98; *p* = 0.025). Loneliness did not significantly affect physical activity in either group. Having more physically active friends was strongly correlated with higher levels of physical activity in both the total and non-isolated groups (total: OR = 1.30; non-isolated: OR = 1.31; both *p* < 0.001), with a trend towards significance in the isolated group (OR = 1.22; *p* = 0.066).

Nagelkerke R² values showed that the non-isolated group had a slightly higher value (0.218) than the isolated group (0.180). The chi-square test confirmed the significance of the model fit, indicating robust findings across the variables.

### 3.5. Comparing the Determinants of Physical Activity between Types of Social Isolation

The impact of different types of social isolation on the likelihood of participating in physical activity (defined as engaging in physical activity for at least one hour per day, three times per week) was examined (Table 5). The participants were categorized into three groups based on their type of isolation: socially avoidant (*n* = 701), socially disinterested (*n* = 435), or actively isolated (*n* = 525).

For all types of social isolation, girls were significantly less likely to participate in physical activity than boys. The ORs were 0.41 for socially avoidant, 0.26 for socially disinterested, and 0.47 for actively isolated (all *p* < 0.001). High school students were less likely to engage in physical activity than middle school students for all types of isolation, with statistically significant differences noted. Adolescents who rated their health as above average were more likely to engage in physical activity, particularly in the actively isolated group (OR = 1.91; *p* = 0.006). There was no significant association between household economic status and physical activity for any group. The effect of loneliness on physical activity was borderline significant in the actively isolated group (*p* = 0.050). In the actively isolated group, having more physically active friends significantly influenced participation in physical activity (OR = 1.42; *p* = 0.020).

Nagelkerke R^2^ values indicate the explanatory power of the models, with the highest value for the actively isolated group (0.230). Chi-square tests confirmed the statistical significance of the models and demonstrated robust findings across the variables.

## 4. Discussion

The purpose of this study was to investigate the relationship between social isolation and physical activity among adolescents and to analyze the differences in factors affecting physical activity according to the types of social isolation. The study found that adolescents who are socially isolated tend to be less physically active than those who are not isolated. Additionally, the impact on physical activity varied depending on the specific type of social isolation. Disinterested and socially avoidant isolation were associated with lower physical activity, while non-isolated and actively isolated adolescents were more active. The factors influencing physical activity also differed according to the specific type of isolation. These findings indicate that different types of social isolation have impacts on physical activity levels, underscoring the importance of considering the multidimensional nature of social isolation in health behavior research.

The study confirms that socially isolated adolescents are generally less active than their non-isolated peers. This finding aligns with the results of previous studies suggesting that social isolation can negatively impact physical activity levels because of a lack of peer support and encouragement [35,41].

Additionally, the results show significant differences in physical activity levels among various types of social isolation. This suggests that motivations and barriers to physical activity differ across isolation types, necessitating tailored intervention strategies. Socially avoidant adolescents who neither nominate nor are nominated by peers as friends, had the lowest levels of physical activity. Previous research conducted on Chinese children [42] also indicated that avoidant children tended to prefer sedentary activities over physical ones, citing a lack of social motivation as a significant influencing factor. Previous studies [42,43] have noted that children who engage more frequently in sedentary behaviors, such as gaming or watching TV, tend to spend more time on these activities. Furthermore, it has been suggested that such sedentary behaviors are often used as a means to alleviate social anxiety, loneliness, or stress caused by social isolation, ultimately leading to a lack of physical activity. Therefore, it is essential to implement efforts to improve the mental health of socially avoidant adolescents to encourage greater participation in physical activities and the formation of healthy social relationships.

In contrast, socially disinterested adolescents are those who are nominated by peers but do not nominate others in return. This group lacked interest in forming reciprocal social bonds, resulting in a lack of social motivation for physical activity. Systematic reviews on factors that promote or hinder physical activity among adolescents [38,44] have reported that low levels of interaction reduce intrinsic motivation, thereby impeding the practice of physical activity. Studies examining intrinsic motivation and physical activity in adolescents for obesity treatment [45] have also supported this finding. Furthermore, socially disinterested adolescents typically preferred to spend time alone rather than engaging in activities with friends, which made them less likely to participate in school or community programs [45]. Therefore, tailored interventions are required to stimulate intrinsic motivation in socially disinterested adolescents by actively promoting the benefits of physical activity and strengthening social support systems.

Interestingly, actively isolated adolescents who attempted to form friendships but did not receive reciprocal nominations had slightly higher levels of physical activity than other isolated groups. These adolescents have a strong motivation to form and maintain relationships with their peers and were relatively more inclined to engage in physical activities as a means of building social connections [46,47]. However, stress from repeated social rejection can also lead to unhealthy behaviors, highlighting the need for interventions that consider the complex interplay between social dynamics and health behaviors [46,48].

The gender gap in physical activity levels, with girls being less active than boys across all isolation types, aligns with the global trends reported by the WHO [6]. This gender disparity underscores the need for interventions specifically aimed at encouraging physical activity in socially isolated girls.

The study also found that high school students, particularly those who are socially isolated, participate less in physical activities than middle school students, which is consistent with existing research findings [49]. This highlights the trend of decreasing activity levels with increasing age and indicates the need for interventions that account for the changing social and academic pressures associated with different age groups.

This study found that subjective health status was an important factor in promoting physical activity across all groups, indicating that adolescents who perceived their health positively were more likely to be active [50,51]. This suggests that even during the relatively healthy period of adolescence, efforts to improve health perceptions can enhance confidence and motivation to engage in physical activities.

Although loneliness was not a significant predictor of physical activity in this study, the borderline significance in the actively isolated group suggests that the emotional experience of loneliness may still play a role in health behaviors. Future studies should explore, more deeply, the potential mediating effects of loneliness on the relationship between social isolation and physical activity.

The number of friends who engaged in physical activity was a strong predictor of physical activity levels, particularly in the non-isolated and actively isolated groups, which is consistent with existing research findings [12]. This underscores the significant influence of peer support in promoting an active lifestyle and suggests that interventions that encourage peer participation could be beneficial.

One of the limitations of this study’s methodology is the partial reliance on survey tools that have not undergone separate validation, potentially impacting the reliability of the results. The survey tool employed to measure the happiness index included only a subset of designed instruments. Additionally, while key variables such as physical activity, isolation status, and types of isolation were defined based on existing research, most variables relied on simple self-report items. Despite the clear articulation of survey questions for each variable, the absence of systematic measurement underscores the need for future research to employ more robustly validated tools to improve the validity and reliability of the findings. Recognizing these limitations is essential when interpreting the results, and further investigation is necessary to enhance the study’s overall rigor.

Additionally, this study measures physical activity using self-reported surveys. However, systematic reviews [52] reveal inconsistent correlations between these surveys and accelerometers, highlighting the need for their combined use to achieve more accurate measurements. Future research should therefore equip adolescents with devices such as accelerometers to enhance the precision of physical activity measurements. This method will not only improve result accuracy but also deepen our understanding of the relationship between physical activity and isolation. Interpreting the results of this study presents challenges. This is due to the scarcity of research on the types of social isolation and physical activity among adolescents, leading us to primarily cite observational and intervention study results. These studies were conducted under different conditions from our own, which may not align with the context of this study and could make establishing causality difficult. Additionally, the applicability of intervention study outcomes is limited, potentially affecting the reliability of our findings. Recognizing these limitations is essential, and future research should use more systematic and validated data.

Given the significant gender differences observed, further studies are required to investigate gender-specific barriers and facilitators of physical activity among socially isolated adolescents. Moreover, qualitative studies could offer valuable perspectives on the lived experiences of isolated adolescents to assist in individualizing interventions to their specific needs.

Future studies should consider longitudinal designs to better understand the causal relationships among social isolation, loneliness, and physical activity. Additionally, exploring the mechanisms by which different types of social isolation affect health behaviors can provide deeper insights into effective intervention strategies.

## 5. Conclusions

The study aimed to examine the relationship between social isolation and physical activity among adolescents, focusing on how different types of isolation impact activity levels. The findings indicate that socially isolated adolescents are generally less active than their non-isolated peers. Specifically, disinterested and socially avoidant adolescents showed lower physical activity, while actively isolated and non-isolated adolescents were more active.

These results suggest the need for tailored interventions that address the unique motivations and barriers faced by different types of socially isolated adolescents. Promoting physical activity among isolated adolescents requires strategies that consider their specific social contexts and psychological needs.

## Figures and Tables

**Table 1:** General characteristics of participants (N = 3356).

Analyzed Variables		N	Percentage or Mean (±SD)
Gender	Boy	1600	47.7
	Girl	1756	52.3
Age	13	525	15.6
	14	472	14.1
	15	655	19.5
	16	666	19.8
	17	562	16.7
	18	476	14.2
School level	Middle school	1652	49.2
	High school	1704	50.8
Self-rated health	Below average (≤3)	817	24.3
	Above average (≥4)	2536	75.6
Household subjective economic status	1–6, where a lower score indicates greater wealth	3318	3.3 (0.9)
Loneliness	Not lonely	2826	84.2
	Lonely	526	15.7
Number of physically active friends		3356	0.50 (1.0)
Social isolation sub-type	Sociable, non-isolated	1695	50.5
	Socially avoidant	701	20.9
	Socially disinterested	435	13.0
	Actively isolated	525	15.6
Physical Activity	Fewer than 3 times per week	1734	51.7
	3 or more times per week	1577	47.0

**Table 2:** Comparison of physical activity based on social isolation status.

Analyzed Variables		Total		Isolation Status				
		N	%	Sociable, Non-Isolated		Isolated		χ^2^ (*p*)
				N	%	N	%	
Physical Activity	Fewer than 3 times per week	1734	52.4	827	49.3	907	55.5	13.0 (<0.001)
	3 or more times per week	1577	47.6	851	50.7	726	44.5	
	Total	3311	100.0	1678	100.0	1633	100.0	

**Table 3:** Comparison of physical activity by types of social isolation.

		Total		Social Isolation Sub-Type								
		N	%	Sociable, Non-Isolated		Socially Avoidant		Socially Disinterested		Actively Isolated		χ^2^ (*p*)
				N	%	N	%	N	%	N	%	
Physical Activity	Fewer than 3 times per week	1734	52.4	827	49.3	402	58.7	235	54.8	270	52.0	18.3 (<0.001)
	3 or more times per week	1577	47.6	851	50.7	283	41.3	194	45.2	249	48.0	
	Total	3311	100.0	1678	100.0	685	100.0	429	100.0	519	100.0	

**Table 4:** Logistic regression analysis comparing the determinants of physical activity between socially isolated and non-isolated Korean adolescents.

Analyzed Variables		Total (N = 3311)				Sociable, Non-Isolated (N = 1678)				Isolated (N = 1633)			
		OR	95% CI		*p*	OR	95% CI		*p*	OR	95% CI		*p*
Gender	Boy (ref)												
	Girl	0.42	0.36	0.49	<0.001	0.46	0.37	0.57	<0.001	0.38	0.31	0.47	<0.001
School level	Middle (ref)												
	High	0.54	0.41	0.72	<0.001	0.72	0.47	1.10	0.129	0.42	0.28	0.63	<0.001
Age		0.86	0.79	0.94	0.001	0.78	0.68	0.89	<0.001	0.95	0.83	1.08	0.403
Self-rated health	Below average (≤3, ref)												
	Above average (≥4)	1.69	1.41	2.02	<0.001	1.70	1.31	2.20	<0.001	1.67	1.30	2.15	<0.001
Household subjective economic status (a lower score indicates greater wealth)		0.93	0.85	1.01	0.088	0.99	0.88	1.12	0.914	0.87	0.78	0.98	0.025
Loneliness	Not lonely (ref)												
	Lonely	0.95	0.77	1.18	0.657	0.90	0.67	1.21	0.488	0.99	0.74	1.33	0.967
Number of physically active friends		1.30	1.18	1.42	<0.001	1.31	1.18	1.46	<0.001	1.22	0.99	1.51	0.066
−2 log likelihood		3994.07				1999.05				1986.79			
Nagelkerke R^2^		0.201				0.218				0.180			
Chi-square (df)		533.18 (7)				294.89 (7)				233.37 (7)			

Note: ref indicates reference. In the logistic regression model, 3311 respondents who answered the physical activity items out of the total sample of 3356 respondents were included in the analysis.

**Table 5:** Comparative logistic regression analysis of physical activity influences among Korean adolescents by types of social isolation.

Analyzed Variables			Socially Avoidant (N = 701)				Socially Disinterested (N = 435)				Actively Isolated (N = 525)			
			OR	95% CI		*p*	OR	95% CI		*p*	OR	95% CI		*p*
Gender	Boy (ref)													
	Girl		0.41	0.29	0.56	<0.001	0.26	0.17	0.40	<0.001	0.47	0.32	0.70	<0.001
School level	Middle (ref)													
	high		0.51	0.28	0.93	0.029	0.29	0.13	0.66	0.003	0.44	0.21	0.94	0.034
Age			0.93	0.76	1.15	0.505	1.02	0.79	1.31	0.899	0.90	0.72	1.14	0.392
Self-rated health	Below average (≤3, ref)													
	Above average (≥4)		1.65	1.13	2.42	0.010	1.57	0.96	2.57	0.075	1.91	1.20	3.04	0.006
Household subjective economic status (a lower score indicates greater wealth)			0.84	0.70	1.01	0.059	0.98	0.78	1.23	0.862	0.83	0.67	1.03	0.095
Loneliness		Not lonely (ref)												
		Lonely	1.23	0.79	1.90	0.353	1.23	0.72	2.10	0.440	0.53	0.28	1.00	0.050
Number of physically active friends			N/A				0.91	0.62	1.35	0.639	1.42	1.06	1.92	0.020
−2 Log likelihood			848.52				508.92				612.05			
Nagelkerke R^2^			0.138				0.216				0.230			
Chi-Square (df)			73.476 (6)				74.700 (7)				96.946 (7)			

Note: ref indicates reference. In the logistic regression model, 3311 respondents who answered the physical activity items out of the total sample of 3356 respondents were included in the analysis. For the logistic regression model analyzing the avoidant isolated type, there were no respondents with data on the number of physically active friends. As a result, the beta value and *p*-value for this variable are marked as “N/A” (not applicable).

## Data Availability

We kindly request that any inquiries regarding the data used in this study be directed to the corresponding author. The data sets used in this study are available upon request.
